# Antioxidant and Antiproliferative Activity of the Ethanolic Extract of *Equisetum myriochaetum* and Molecular Docking of Its Main Metabolites (Apigenin, Kaempferol, and Quercetin) on β-Tubulin

**DOI:** 10.3390/molecules26020443

**Published:** 2021-01-16

**Authors:** Fabián Olazarán-Santibañez, Gildardo Rivera, Venancio Vanoye-Eligio, Arturo Mora-Olivo, Gabriel Aguirre-Guzmán, Mónica Ramírez-Cabrera, Eder Arredondo-Espinoza

**Affiliations:** 1 Instituto de Ecología Aplicada, Universidad Autónoma de Tamaulipas, Ciudad Victoria 87019, Mexico; vvanoye@docentes.uat.edu.mx (V.V.-E.); amorao@docentes.uat.edu.mx (A.M.-O.); 2 Centro de Biotecnología Genómica, Laboratorio de Biotecnología Farmacéutica, Instituto Politécnico Nacional, Reynosa 88710, Mexico; gildardors@hotmail.com; 3 Facultad de Medicina Veterinaria y Zootecnia, Universidad Autónoma de Tamaulipas, Ciudad Victoria 87000, Mexico; gabaguirre@docentes.uat.edu.mx; 4 Laboratorio de Farmacología Molecular y Modelos Biológicos, Facultad de Ciencias Químicas, Universidad Autónoma de Nuevo Leon, Av Guerrero s/n, Treviño, C.P., Monterrey 64570, Mexico; monica.ramirezcbr@uanl.edu.mx (M.R.-C.); eder.arredondosp@uanl.edu.mx (E.A.-E.)

**Keywords:** antioxidative, antiproliferative, *Equisetum myriochaetum*, flavonoids, kaempferol, molecular docking

## Abstract

*Equisetum myriochaetum* is a semi-aquatic plant found on riverbanks that is commonly used in traditional medicine as a diuretic agent. Additionally, the genus *Equisetum* stands out for its content of the flavonoid kaempferol, a well-known antiproliferative agent. Therefore, in this study, *E. myriochaetum* ethanolic extract was tested in vitro against a cervical cancer cell line (SiHa). Additionally, the antioxidative activity was evaluated through a 2,2-diphenyl-1-picrilhidrazil (DPPH) assay. Finally, a molecular docking analysis of apigenin, kaempferol, and quercetin on the active site of β-tubulin was performed to investigate their potential mechanism of action. All fractions of *E. myriochaetum* ethanolic extract showed antioxidative activity. Fraction 14 displayed an antiproliferative capacity with a half maximal inhibitory concentration (IC_50_) value of 6.78 μg/mL against SiHa cells.

## 1. Introduction

The genus *Equisetum* (Equisetaceae) includes 15 species of perennial herbs with a nearly cosmopolitan distribution, which mostly inhabit aquatic environments or swamps [1,2]. The species *E. hyemale* (L.) and *E. myriochaetum* (Schltdl. & Cham.) (known commonly as horsetails) are widely distributed in Mexico, where their medicinal properties are associated with their consumption [3,4]. The medicinal effects associated with this plant include antimicrobial, antioxidant, antiproliferative, and diuretic properties [5,6,7,8]. The antiproliferative capacity of these species has been limited to *E. arvense* (L.), whose ethanolic extracts showed a cytotoxic effect in the lung carcinoma cell line A549 [9]. Phytochemical analyses of *E. arvense* showed the presence of saponins, alkaloids, triterpenoids, and flavonoids, which could support its wide spectra of bioactive effects, including its antiproliferative potential [10].

A phytochemical study of hydroethanolic extracts from *E. giganteum* (L.) showed the presence of flavonoid derivates (mainly quercetin and kaempferol) and presented significant anti-inflammatory, antioxidant, and antiproliferative activities [11]. Similarly, an analysis of aqueous extracts from *E. myriochaetum* showed the presence of flavonoid derivatives, such as kaempferol-3-*O*-sophoroside, kaempferol-3,7-di-*O*-β-glucoside, kaempferol-3-*O*-sophoroside-4’-*O*-β-glucoside, and caffeoylmethylate-4-β-glucopyranoside [12]. Aqueous and butanoic extracts from *E. myriochaetum* did not show a genotoxic effect, but did, however, show a hypoglycemic effect in patients with type 2 diabetes after 90 min of application [13].

In vivo tests of the aqueous extract of *E. myriochaetum* showed an inhibitory effect on the production of hepatic glucose at 120 min with 324 mg/dL during the pyruvate tolerance test when using metformin as a hypoglycemic control [14]. This evidence suggests the biological potential hypoglycemia of these species as a whole; however, *E. myriochaetum* has been hardly studied as a reservoir of additional bioactive chemical compounds, such as antiproliferative agents, even when some related *Equisetum* species showed this potential [15,16].

Cervical cancer, as a gynecological tumor, remains one of the most common causes of cancer-related deaths worldwide [17]. Thus, there is an urgent need to develop novel treatments for cervical cancer. Therefore, the main aim in this study was a bio-directed extraction of flavonoids from *E. myriochaetum* to evaluate their antioxidant activity. After that, a phytochemical characterization by Ultra-Performance Liquid Chromatography–Mass Spectrometry (UPLC–MS) was performed to obtain the main secondary metabolites and the antiproliferative activity was analyzed in silico and in vitro against a cervical cancer cell line (SiHa). Finally, molecular docking of the main metabolites on the active site of β-tubulin was used to investigate the potential mechanism of action.

## 2. Results

### 2.1. Total Phenolic Compounds and Antioxidant Capacity

In the present study, the bio-directed extraction of phenolic compounds from *E. myriochaetum* was carried out through different organic solvents. Table 1 shows the results of the quantification of the total phenolic compounds in the four crude extracts of hexane, dichloromethane, ethanol, and water. The crude ethanol extract presented the highest concentration of total phenolic compounds expressed in gallic acid equivalents (15.51 GAE mg/g). Therefore, the ethanolic extract of *E. myriochaetum* was chosen for its subsequent fractionation and studies.

The ethanolic extract of *E. myriochaetum* was separated by column chromatography into 16 fractions, which were evaluated for their antioxidant capacity (Table 2). Fraction 14 presented the highest value equivalent to Trolox (423.16 mM TE/g). For this reason, we chose to analyze its chemical composition by Ultra-Performance Liquid Chromatography (UPLC).

### 2.2. Ultra-Performance Liquid Chromatography Analysis and Analysis In Silico

**Figure 1:**
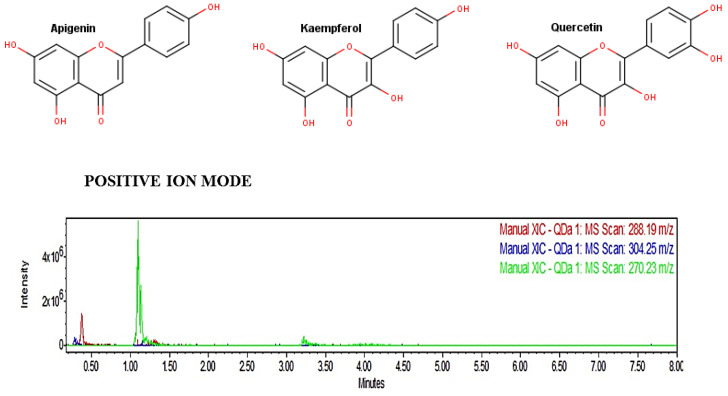
Chemical structure of the flavonoids kaempferol (288 g/mol), quercetin (304 g/mol), and apigenin (270 g/mol), detected in fraction 14 by Ultra-Performance Liquid Chromatography–Mass Spectrometry (UPLC–MS).

The UPLC analysis showed that fraction 14 of the ethanol extract of *E. myriochaetum* contained the flavonoids apigenin, kaempferol, and quercetin (Figure 1). Therefore, the possible pharmacological activities of flavonoids were analyzed using the Prediction of Activity Spectra for Substances (PASS) software.

The predicted pharmacological activities for the metabolites kaempferol, quercetin, and apigenin are presented in Table 3. Additionally, other predictions of pharmacological activity are presented in the Supplementary Materials.

### 2.3. Antiproliferative Activity and Molecular Docking

Based on the pharmacological prediction, the antiproliferative activity of fraction 14 was evaluated against the cervical cancer cell line (SiHa). Fraction 14 showed an antiproliferative effect against SiHa cells in a concentration-dependent manner. Concentrations of 200–3175 μg/mL were able to inhibit proliferation in 50% of the cells compared to the untreated control. A linear regression analysis of the dose–response curve determined an half maximal inhibitory concentration (IC_50_) of 6.78 ± 0.6 μg/mL. The cytostatic drug paclitaxel presented an IC_50_ value of 0.364 ± 0.03 μg/mL.

To know the potential mechanism of action of the metabolites kaempferol, quercetin, and apigenin, found in fraction 14, a bibliographic search and an analysis in SwissTarget Prediction was performed (http://www.swisstargetprediction.ch/) [18]. The results suggested β-tubulin as a potential pharmacological target and, therefore, a molecular docking analysis was carried out. Table 4 shows the binding energy values of kaempferol, quercetin, and apigenin on the reported active sites of β-tubulin, as well as the values of the reference drugs colchicine, paclitaxel, and vinblastine.

**Figure 2:**
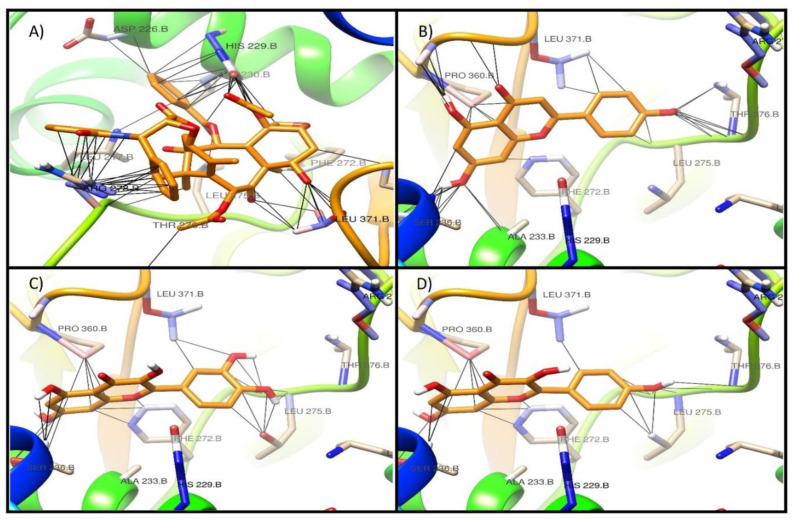
Interaction of the main metabolites of fraction 14 of the ethanol extract of *E. myriochaetum* on the active site of taxol in β-tubulin. (**A**) paclitaxel, (**B**) apigenin, (**C**) quercetin, (**D**) kaempferol.

**Figure 3:**
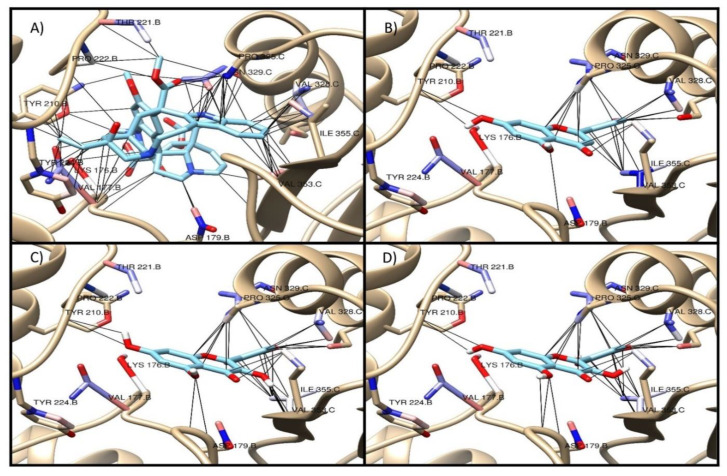
Interaction of the main metabolites of fraction 14 of the ethanol extract of *E. myriochaetum* on the active site of vinca in β-tubulin. (**A**) vinblastine, (**B**) apigenin, (**C**) quercetin, (**D**) kaempferol.

**Figure 4:**
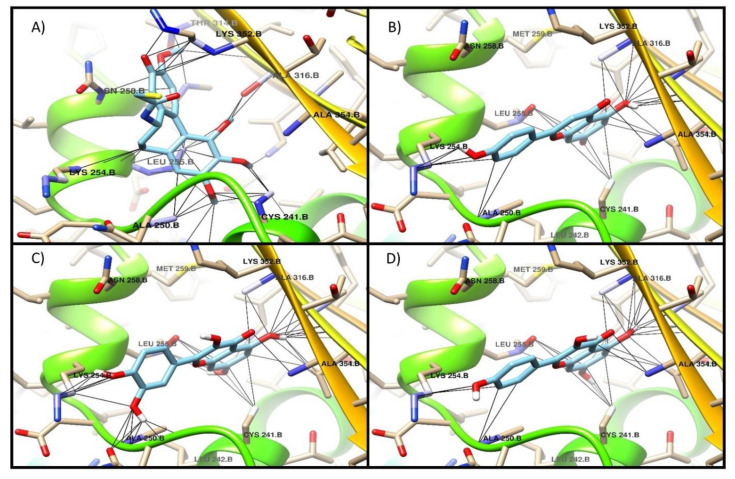
Interaction of the main metabolites of fraction 14 of the ethanol extract of *E. myriochaetum* on the active site of colchicine in β-tubulin. (**A**) colchicine, (**B**) apigenin, (**C**) quercetin, (**D**) kaempferol.

Additionally, Figure 2, Figure 3 and Figure 4 show the interaction profile of the metabolites quercetin, kaempferol, and apigenin on the active sites of β-tubulin reported for taxol, vinca, and colchicine.

## 3. Discussion

In this study, a bio-directed extraction was conducted to obtain mainly secondary metabolites of the flavonoid type, using hexane, dichloromethane, ethanol, and water. As expected, the ethanol extract had the highest quantity of phenolic compounds [19]. However, the other extracts also presented phenolic compounds due to the great diversity of this type of metabolite in plants [20].

Based on the above, the ethanol extract was selected for its fractionation and the subsequent determination of its antioxidant capacity. The results showed high equivalent levels of Trolox in fractions 14 and 16 with values greater than 400, and 11 more fractions presented values greater than 300, but less than 400, and the rest of the fractions presented values less than 300. The level of activity against 2,2-diphenyl-1-picrilhidrazil (DPPH) has typically been considered high for crude extracts from *Equisetum* species using several solvents, including ethanol and water [21,22,23]; however, the quantity detected in this work was considerable after fractionation and concentration procedures.

Analysis of the fraction by UPLC–MS allowed for the detection of three phenolic compounds: kaempferol, quercetin, and apigenin. Figure 1 shows a high relative concentration of apigenin but not quercetin or kaempferol. Subsequently, in silico analysis for the prediction of the biological activity of these metabolites (PASS) indicated that they may have an antioxidant and antiproliferative activity (Pa value > 0.7). Commonly, in drug design, a Pa value greater than 0.7 equates to a high prediction of activity. Even tough, some authors indicate that values greater than 0.5 could indicate a good prediction [24,25,26]. Additionally, previous reports showed that these flavonoids have biological activities—for example, as pro-apoptotic, antioxidants, and antiproliferative agents [27,28,29].

In the in vitro analysis against the SiHa cell line, fraction 14 presented an antiproliferative activity lower than that presented by paclitaxel. This suggests that the activity of fraction 14 may be related to the secondary metabolite apigenin, which was present at a higher concentration. Previous reports indicated that apigenin had favorable effects against carcinogenesis, metastasis, and angiogenesis by stimulating cell death by apoptosis or autophagy [30].

In this regard, Tong et al. (2012) reported the chemopreventive activity of apigenin in human keratinocytes by inducing autophagy through the activation of activated protein kinase or AMP-activated protein kinase (AMPK) [31]. Additionally, the molecular docking study showed that the metabolites quercetin, kaempferol, and apigenin have affinity on the active sites of paclitaxel and vinca, with a binding energy value −7 kcal/mol, although this value is lower than that of the reference drugs at both sites [32,33].

However, apigenin, and kaempferol showed better binding energy values than the reference drug of the active site of colchicine, which suggests that this could be their mechanism of action to exert an antiproliferative effect [34]. Phytochemical exploration and characterization of the extracts could lead to the discovery of the potential bioactive properties of medicinal plants, and this principle coincides with findings in this work, where the antioxidative selection of fractions allowed for further assays [35,36,37].

## 4. Materials and Methods

### 4.1. Collection and Identification of E. Myriochaetum

The specimens of *E. myriochaetum* were collected from the banks of the San Felipe river within the Ecological Park “Los Troncones”, which is located in the northern section of Ciudad Victoria, Tamaulipas, Mexico (23°46’42.0”N 99°12’25.3”W). Parts of the collected specimens were pressed, identified, and stored in the herbarium of the Instituto de Ecología Aplicada, Universidad Autónoma de Tamaulipas, with voucher number 022753 [38]. The remaining specimens were washed with distilled water and dried at 40 °C for 72 h in a hot air oven (Lab Companion ON-12G; Korea). The extracts were prepared by passing different solvents (hexane, dichloromethane, ethanol, and distilled water CTR Scientific, México) through 100 g of *E. myriochaetum* in one liter of solvent in the dark, at room temperature, for 5 days. After this, the solution was filtrated and concentrated in a rotary evaporator until reaching a solid state at 40 °C (RE100-Pro, DLAB Scientific Inc., Ontario, California, USA).

### 4.2. Total Phenolic Compounds and Antioxidative Capacity

The total phenolic compounds were quantified using the Folin–Ciocalteu method [39]. A standard curve from 1 to 8 μg/mL of gallic acid was used. An aliquot of 10 μL (of the sample or standard) was mixed with 25 μL of Folin–Ciocalteu reagent and 125 μL of 2M Na_2_CO_3_ and incubated for 2 h at room temperature (20 ± 5 °C). The absorbance of samples and standards was recorded at 760 nm. The results were expressed in gallic acid equivalents (GAE mg/g). The ethanolic extract was fractioned with 16 fractions by column chromatography using a silica gel pore size of 60 Å, a 230–400 mesh particle size as the stationary phase and hexane/ethanol as the mobile phase with a concentration gradient from 100 to 0% hexane (Hexane RA, CTR Scientific, Monterrey, Nuevo León, Mexico). After this, the fraction was evaporated, and the dry extract was stored at −20 °C [40].

The inhibition of the radical 2,2-diphenyl-1-picrilhidrazil (DPPH) was calculated using 25 μL of the sample fraction (2 mg/mL) or a standard from a Trolox standard solution (Sigma-Aldrich, St. Louis MO, USA), which were mixed with 975 μL of 600 µM DPPH dissolved in methanol (λ = 0.7 at 515 nm). After 30 min in the dark, the absorbance of samples was recorded at 515 nm using a UV/Vis spectrophotometer (UV-6000 UV/VIS Spectrophot, Metash; China). The plotting of the absorbance values of the Trolox standard into a concentration curve was used to determine the concentration of the samples as µmol of Trolox per grams of extract (TE/g) [41].

### 4.3. Flavonoid Detection

Five mg of fraction 14 with the highest antioxidative activity were dissolved in 0.9 mL of methanol and analyzed in a UPLC coupled with an ACQUITY QDa mass detector (Waters, MA USA). The following conditions were used: ACQUITY UPLC^®^ CORTECS^®^ C^18^ column (1.6 µm, 3.0 × 100 mm); mobile phase A (0.1% formic acid) and B (100% acetonitrile) in gradient run (0.5 min, 10.0% A, 90.0% B; 3.5 min, 50.0% A, 50.0% B; and 6 min, 10.0% A, 90.0% B), total time of execution: 6.0 min; caudal: 0.3 mL/min; injection volume: 2 µL; and column temperature: 40 °C. The identification of the main compounds was performed using a range of phenolic compounds, including kaempferol, quercetin, and apigenin (Sigma-Aldrich, St. Louis, MO, USA) [42].

### 4.4. Prediction of Biological Activity In Silico

The flavonoid-derived compounds from *E. myriochaetum* were analyzed in silico using the Prediction of Activity Spectra for Substances (PASS^®^) v2017 software (available to download at http://www.pharmaexpert.ru/passonline/). The molecular structure was predicted through this program, thus determining the compounds’ biological probability of being active (Pa) or inactive (Pi) [43].

### 4.5. Antiproliferative Activity

The cervical cancer cell line SiHa (ATCC^®^ HTB-35™) was cultured in Dulbecco’s Modified Eagle Medium (DMEM) supplemented with 10% Fetal Bovine Serum (FBS). Approximately 5000 cells were seeded per well in 96-well plates and incubated at 37 °C/5% CO_2_. After 24 h, the cells were supplemented with the highest antioxidative fraction of *E. myriochaetum* (diluted in MiliQ water at a concentration of 2000 µg/mL). From this solution, dilutions were prepared (200, 100, 50, 25, 12.5, 6.25, and 3.17 µg/mL).

After 48 h, the media were replaced with 100 µL of fresh media supplemented with 10 µL WST-1 [2-(4 iodophenyl)-3-(4-nitrofenil)-5-(2,4-disulfofenil)-2*H*-tetrazolio] (Merck, Darmstadt; Germany) and incubated for 2 h. The optical density was measured at 450 nm using a Universal Microplate Reader ELx800 (Biotek Instrument, Winooski, Vermont; USA). The WST-1 test determined the viability of the cells, thus allowing a linear regression analysis on the dose response curve in order to determine the IC_50_ of the samples and compare these values against the cytostatic paclitaxel [44]. The statistical analyses were done using the GraphPad Prism software, version 7 (GraphPad, La Jolla, CA, USA).

### 4.6. Molecular Docking

Analysis in Swiss Target Prediction [18]: The β-tubulin models 1SA0, 1FJJ, and 1Z2B were obtained from the Protein Data Bank database (PDB) [45]. The structures of the flavonoid compounds detected by UPLC analysis were drawn with the Marvin ChemAxon software [46], and their energy minimization was calculated with the Avogadro software [47]. The obtained data were converted into an AutoDock format (pdbqt) [48], and the values of the chemical interaction energy for the different binding sites were obtained and visualized with the UCSF Chimera software (available to download at https://www.cgl.ucsf.edu/chimera/) [49].

### 4.7. Statistical Analysis

The antioxidative capacity results were expressed as the standard error of the mean in μmol Trolox per grams of extract (TE/g) at each time point on the curve. One-way ANOVA with Tukey’s test were performed to compare the mean Trolox equivalents between all groups at each time point, the mean Trolox equivalents of each group against the corresponding baseline values, and the Area Under The Curve (AUC) values between groups. *p* values less than 0.05 were considered statistically significant. The statistical analyses used GraphPad Prism software, version 7 (GraphPad, La Jolla, CA, USA).

## 5. Conclusions

Biodirected extraction of the phenolic compounds from *E. myriochaetum* made it possible to isolate a fraction with a high content of equivalent units of Trolox. Additionally, fraction 14 presented antiproliferative activity with an IC_50_ value of 6.78 μg/mL. This fraction presented the secondary metabolites kaempferol, quercetin, and apigenin, which may be responsible for its antioxidant and antiproliferative activity. Molecular docking analysis suggested that the mechanism of action of these metabolites is through their preferential binding to the active site of colchicine. This confirms the importance of achieving the identification of the metabolites responsible for biological activity and their potential mechanisms of action, which may allow for the development of new pharmacological agents.

## Figures and Tables

**Table 1:** Total phenolic compounds (TPC) by the Folin–Ciocalteu method and yield extracted in different solvents (hexane, dichloromethane, ethanol, and water) of *E. myriochaetum*.

Solvents	TPC (GAE mg/g)	Yield Extracted (%)
Hexane	0.91 ± 0.33	5.7
Dichloromethane	2.43 ± 0.58	1.2
Ethanol	15.51 ± 0.99	7.7
Water	11.80 ± 0.39	4.1

**Table 2:** The antioxidant capacity of the fractions of the ethanol extract of *E. myriochaetum*, determined with 2,2-diphenyl-1-picrilhidrazil (DPPH) expressed in Trolox equivalents per gram of sample (mM TE/g).

Fraction	Gradient (% H/% Et) *	Yield Extracted (%)	DPPH (mM TE/g)
1	100/0	0.9	313.51 ± 3.16
2	95/5	0.6	315.22 ± 1.48
3	90/10	1.2	316.29 ± 1.11
4	85/15	2.3	295.59 ± 2.03
5	80/20	12.7	254.84 ± 8.96
6	75/25	5.5	320.55 ± 1.84
7	70/30	1.6	293.03 ± 9.32
8	65/35	0.6	341.24 ± 10.76
9	60/40	2.7	327.38 ± 1.61
10	55/45	2.0	330.36 ± 2.93
11	50/50	2.2	337.40 ± 1.10
12	40/60	0.8	347.43 ± 8.59
13	30/70	0.3	341.46 ± 5.44
14	20/80	1.4	423.16 ± 4.19
15	10/90	1.3	376.02 ± 2.05
16	0/100	0.5	406.31 ± 0.97

* H: Hexane; Et: Ethanol.

**Table 3:** Biological activity prediction by Prediction of Activity Spectra for Substances (PASS) software.

Compound	Type of Activity	Pa *	Pi *
Kaempferol	Antioxidant	0.864	0.003
	Antiproliferative	0.720	0.008
Quercetin	Antioxidant	0.878	0.003
	Antiproliferative	0.761	0.007
Apigenin	Antioxidant	0.740	0.004
	Antiproliferative	0.775	0.015

* Pa: Probability of being active, and Pi: Probability of being inactive.

**Table 4:** Binding energy of flavonoids (apigenin, kaempferol, and quercetin) on the active site of β-tubulin by molecular docking.

Compound	Taxol Site, Vina Score (Kcal/Mol)	Vinca Site, Vina Score (Kcal/Mol)	Colchicine Site, Vina Score (Kcal/Mol)
Apigenin	−7.3	−7.1	−7.6
Kaempferol	−7.1	−7.3	−7.4
Quercetin	−7.2	−7.2	−7.1
Paclitaxel *	−9.8	--	--
Vinblastine **	--	−10	--
Colchicine ***	--	--	−7.4

* Control Taxol site PDB (1JFF). ** Control Vinca site PDB (1Z2B). *** Control Colchicine site PDB (1SA0)

## Data Availability

The data presented in this study are available on request from the corresponding author.

## Supplementary Materials

The following are available online, Table S1: apigenin, kaempferol, quercetin.

## Sample Availability

Samples of the compounds (apigenin, kaempferol, quercetin) are available from the authors.
