# Supplementary material for: Antioxidant and Antiproliferative Activity of the Ethanolic Extract of Equisetum myriochaetum and Molecular Docking of Its Main Metabolites (Apigenin, Kaempferol, and Quercetin) on β-Tubulin
Source: Molecules. 2021 Jan 16;26(2):443. doi: 10.3390/molecules26020443 (PMC7831036; doi:10.3390/molecules26020443)
Supplement: Supplementary file 1 [file molecules-26-00443-s001.pdf]

Apigenin

PA	PI	Activity
0,818	0,001	Testosterone 17beta-dehydrogenase inhibitor
0,819	0,003	Monophenol monooxygenase inhibitor
0,820	0,005	Alkane 1-monooxygenase inhibitor
0,813	0,004	CYP2B5 substrate
0,810	0,004	CYP1A inhibitor
0,809	0,004	UGT1A substrate
0,802	0,003	UGT1A3 substrate
0,801	0,007	UDP-glucuronosyltransferase substrate
0,795	0,003	Leukotriene-B4 20-monooxygenase inhibitor
0,798	0,008	JAK2 expression inhibitor
0,791	0,003	Histamine release inhibitor
0,785	0,004	UGT1A1 substrate
0,782	0,002	1-Alkylglycerophosphocholine O-acetyltransferase inhibitor
0,783	0,004	CYP1A2 inhibitor
0,783	0,003	Pectate lyase inhibitor
0,797	0,019	Mucomembranous protector
0,787	0,011	Dehydro-L-gulonate decarboxylase inhibitor
0,780	0,004	MMP9 expression inhibitor
0,773	0,003	UGT1A7 substrate
0,778	0,008	CYP3A4 inducer
0,771	0,002	CYP1A1 inhibitor
0,764	0,002	NADPH oxidase inhibitor
0,765	0,004	CYP2A4 substrate
0,775	0,015	Antineoplastic (Antiproliferative)
0,757	0,003	UGT1A10 substrate
0,755	0,003	Xenobiotic-transporting ATPase inhibitor
0,755	0,005	Pin1 inhibitor
0,752	0,002	CF transmembrane conductance regulator agonist
0,758	0,011	Glutathione thiolesterase inhibitor
0,778	0,031	Testosterone 17beta-dehydrogenase (NADP+) inhibitor
0,749	0,004	Tetrahydroxynaphthalene reductase inhibitor
0,748	0,004	Nitrite reductase [NAD(P)H] inhibitor
0,745	0,007	CYP1A2 substrate

0,747	0,009	CYP3A inducer
0,739	0,002	Alcohol dehydrogenase [NAD(P)+] inhibitor
0,750	0,013	Glucan endo-1,6-beta-glucosidase inhibitor
0,731	0,001	Creatine kinase inhibitor
0,732	0,002	NOS2 expression inhibitor
0,740	0,004	Antioxidant
0,729	0,002	CYP19 inhibitor
0,730	0,005	Insulysin inhibitor
0,726	0,002	Iodide peroxidase inhibitor
0,729	0,010	NAD(P)+-arginine ADP-ribosyltransferase inhibitor
0,753	0,033	CYP2J substrate
0,721	0,005	5 Hydroxytryptamine release inhibitor
0,720	0,004	UGT2B12 substrate
0,719	0,004	Free radical scavenger
0,716	0,003	UGT2B15 substrate
0,713	0,003	UGT1A8 substrate
0,741	0,036	Gluconate 2-dehydrogenase (acceptor) inhibitor
0,711	0,008	Ecdysone 20-monooxygenase inhibitor
0,703	0,002	CYP1B1 inhibitor
0,726	0,027	CYP2J2 substrate
0,702	0,003	CYP1B substrate
0,706	0,010	Thioredoxin inhibitor
0,703	0,009	CYP2A6 substrate
0,709	0,025	NADPH peroxidase inhibitor
0,706	0,032	Sugar-phosphatase inhibitor

Kaempferol

PA	PI	Activity
0,983	0,001	Chlordecone reductase inhibitor
0,974	0,002	Membrane integrity agonist
0,969	0,002	HIF1A expression inhibitor
0,965	0,001	2-Dehydropantoate 2-reductase inhibitor
0,961	0,001	Aryl-alcohol dehydrogenase (NADP+) inhibitor
0,959	0,001	P-benzoquinone reductase (NADPH) inhibitor
0,959	0,001	Kinase inhibitor
0,957	0,002	Membrane permeability inhibitor
0,956	0,001	Peroxidase inhibitor
0,951	0,001	Quercetin 2,3-dioxygenase inhibitor
0,948	0,001	Antimutagenic
0,947	0,001	NADPH-ferrihemoprotein reductase inhibitor
0,945	0,002	HMOX1 expression enhancer
0,944	0,002	CYP1A inducer
0,940	0,004	CYP1A substrate
0,931	0,001	2-Dehydropantolactone reductase (A-specific) inhibitor
0,931	0,001	Glycerol dehydrogenase (NADP+) inhibitor
0,933	0,003	CYP1A1 substrate
0,931	0,004	TP53 expression enhancer
0,926	0,001	Cystathionine beta-synthase inhibitor
0,926	0,002	UGT1A6 substrate
0,925	0,001	2-Enoate reductase inhibitor
0,924	0,001	CYP1A1 inducer
0,929	0,006	CYP2C12 substrate
0,923	0,002	Alcohol dehydrogenase (NADP+) inhibitor
0,923	0,004	Ubiquinol-cytochrome-c reductase inhibitor
0,919	0,001	Beta-carotene 15,15'-monooxygenase inhibitor
0,919	0,002	UGT1A9 substrate
0,917	0,001	MAP kinase stimulant
0,905	0,002	NADPH oxidase inhibitor
0,907	0,004	CYP1A2 substrate
0,904	0,002	Histidine kinase inhibitor
0,903	0,003	CYP1A inhibitor

0,894	0,002	Antihemorrhagic
0,894	0,004	Anaphylatoxin receptor antagonist
0,890	0,002	Xenobiotic-transporting ATPase inhibitor
0,889	0,003	CYP1A2 inhibitor
0,887	0,002	UGT1A10 substrate
0,889	0,004	Apoptosis agonist
0,881	0,001	Chalcone isomerase inhibitor
0,871	0,002	CYP1A1 inhibitor
0,868	0,002	AR expression inhibitor
0,866	0,001	Testosterone 17beta-dehydrogenase inhibitor
0,867	0,005	Aldehyde oxidase inhibitor
0,864	0,003	Antioxidant
0,860	0,003	UGT1A substrate
0,858	0,002	UGT1A3 substrate
0,855	0,001	Iodide peroxidase inhibitor
0,852	0,010	Antiseborrheic
0,856	0,018	Aspulvinone dimethylallyltransferase inhibitor
0,838	0,003	UGT1A1 substrate
0,835	0,004	UDP-glucuronosyltransferase substrate
0,823	0,001	Creatine kinase inhibitor
0,824	0,002	CYP19 inhibitor
0,817	0,002	CYP1B1 inhibitor
0,821	0,006	CYP3A4 inducer
0,815	0,001	CYP19A1 expression inhibitor
0,812	0,002	NOS2 expression inhibitor
0,813	0,003	Cardioprotectant
0,808	0,002	1-Alkylglycerophosphocholine O-acetyltransferase inhibitor
0,807	0,005	Vasoprotector
0,798	0,001	Alcohol dehydrogenase [NAD(P)+] inhibitor
0,796	0,002	Hemostatic
0,794	0,005	27-Hydroxycholesterol 7alpha-monooxygenase inhibitor
0,782	0,003	UGT1A8 substrate
0,783	0,004	Lipid peroxidase inhibitor
0,784	0,007	CYP3A inducer
0,777	0,004	APOA1 expression enhancer

0,774	0,003	Free radical scavenger
0,775	0,005	4-Nitrophenol 2-monooxygenase inhibitor
0,772	0,002	Morphine 6-dehydrogenase inhibitor
0,770	0,002	Xanthine oxidase inhibitor
0,762	0,005	CYP2B5 substrate
0,758	0,003	CYP2C8 inhibitor
0,755	0,002	3-Methylbutanal reductase inhibitor
0,754	0,002	UGT2B15 substrate
0,753	0,005	Cholestanetriol 26-monooxygenase inhibitor
0,745	0,002	SULT1A3 substrate
0,738	0,005	MMP9 expression inhibitor
0,745	0,013	JAK2 expression inhibitor
0,721	0,003	Interleukin 4 antagonist
0,722	0,004	Malate dehydrogenase inhibitor
0,722	0,004	UGT2B substrate
0,714	0,001	3-Oxoacyl-[acyl-carrier-protein] synthase inhibitor
0,716	0,004	Monophenol monooxygenase inhibitor
0,720	0,008	Antineoplastic (Antiproliferative)
0,715	0,004	Histamine release stimulant
0,712	0,003	CF transmembrane conductance regulator agonist
0,714	0,005	CYP2A4 substrate
0,727	0,019	Dehydro-L-gulonate decarboxylase inhibitor
0,711	0,004	Pectate lyase inhibitor
0,709	0,004	Leukotriene-B4 20-monooxygenase inhibitor
0,705	0,001	Capillary fragility treatment
0,704	0,001	Glutathione-disulfide reductase inhibitor
0,707	0,004	Beta glucuronidase inhibitor
0,716	0,015	CYP2C substrate
0,738	0,040	Mucomembranous protector
0,709	0,015	Alkane 1-monooxygenase inhibitor
0,723	0,048	Testosterone 17beta-dehydrogenase (NADP+) inhibitor
0,709	0,030	Alkenylglycerophosphocholine hydrolase inhibitor

Quercetin

PA	PI	Activity
0,986	0,001	Chlordecone reductase inhibitor
0,973	0,002	Membrane integrity agonist
0,971	0,001	2-Dehydropantoate 2-reductase inhibitor
0,968	0,001	Aryl-alcohol dehydrogenase (NADP+) inhibitor
0,969	0,002	HIF1A expression inhibitor
0,965	0,001	P-benzoquinone reductase (NADPH) inhibitor
0,962	0,001	Peroxidase inhibitor
0,963	0,002	HMOX1 expression enhancer
0,962	0,002	Membrane permeability inhibitor
0,961	0,001	Antimutagenic
0,959	0,001	CYP1A inducer
0,957	0,001	Kinase inhibitor
0,955	0,001	NADPH-ferrihemoprotein reductase inhibitor
0,946	0,002	UGT1A9 substrate
0,943	0,001	Glycerol dehydrogenase (NADP+) inhibitor
0,944	0,002	UGT1A6 substrate
0,945	0,004	CYP1A substrate
0,942	0,001	CYP1A1 inducer
0,941	0,001	2-Dehydropantolactone reductase (A-specific) inhibitor
0,941	0,001	Cystathionine beta-synthase inhibitor
0,942	0,002	UGT1A10 substrate
0,940	0,003	CYP1A1 substrate
0,939	0,004	TP53 expression enhancer
0,938	0,003	CYP1A inhibitor
0,934	0,001	Quercetin 2,3-dioxygenase inhibitor
0,934	0,001	Beta-carotene 15,15'-monooxygenase inhibitor
0,933	0,001	MAP kinase stimulant
0,933	0,002	NADPH oxidase inhibitor
0,927	0,002	Alcohol dehydrogenase (NADP+) inhibitor
0,920	0,001	Chalcone isomerase inhibitor
0,924	0,007	CYP2C12 substrate
0,919	0,003	CYP1A2 inhibitor
0,916	0,001	CYP1A1 inhibitor

0,909	0,004	CYP1A2 substrate
0,906	0,002	UGT1A3 substrate
0,902	0,001	Iodide peroxidase inhibitor
0,898	0,001	2-Enoate reductase inhibitor
0,900	0,005	Ubiquinol-cytochrome-c reductase inhibitor
0,898	0,003	UGT1A substrate
0,896	0,002	Antihemorrhagic
0,895	0,001	Testosterone 17beta-dehydrogenase inhibitor
0,894	0,002	AR expression inhibitor
0,895	0,002	Histidine kinase inhibitor
0,895	0,004	Apoptosis agonist
0,891	0,001	CYP19 inhibitor
0,894	0,004	Aldehyde oxidase inhibitor
0,886	0,002	Xenobiotic-transporting ATPase inhibitor
0,881	0,001	CYP1B1 inhibitor
0,878	0,003	Antioxidant
0,872	0,002	UGT1A1 substrate
0,871	0,001	Creatine kinase inhibitor
0,875	0,005	Anaphylatoxin receptor antagonist
0,866	0,002	Hemostatic
0,859	0,002	NOS2 expression inhibitor
0,859	0,004	UDP-glucuronosyltransferase substrate
0,851	0,003	Cardioprotectant
0,840	0,001	CYP19A1 expression inhibitor
0,828	0,001	Morphine 6-dehydrogenase inhibitor
0,831	0,005	CYP3A4 inducer
0,828	0,002	UGT1A8 substrate
0,825	0,001	1-Alkylglycerophosphocholine O-acetyltransferase inhibitor
0,835	0,013	Antiseborrheic
0,823	0,002	Xanthine oxidase inhibitor
0,824	0,004	Vasoprotector
0,816	0,002	Free radical scavenger
0,811	0,001	3-Methylbutanal reductase inhibitor
0,811	0,001	Alcohol dehydrogenase [NAD(P)+] inhibitor
0,813	0,003	Lipid peroxidase inhibitor

0,796	0,002	SULT1A3 substrate
0,792	0,003	Monophenol monooxygenase inhibitor
0,817	0,029	Aspulvinone dimethylallyltransferase inhibitor
0,794	0,006	CYP3A inducer
0,789	0,004	Sulfotransferase substrate
0,785	0,001	Xanthine dehydrogenase inhibitor
0,786	0,002	UGT2B15 substrate
0,781	0,001	3-Oxoacyl-[acyl-carrier-protein] synthase inhibitor
0,787	0,009	JAK2 expression inhibitor
0,776	0,004	APOA1 expression enhancer
0,769	0,003	CYP2C8 inhibitor
0,761	0,001	Glutathione-disulfide reductase inhibitor
0,761	0,007	Antineoplastic (Antiproliferative)
0,755	0,005	CYP2B5 substrate
0,754	0,005	4-Nitrophenol 2-monooxygenase inhibitor
0,753	0,004	Pectate lyase inhibitor
0,751	0,003	Histamine release stimulant
0,744	0,001	Capillary fragility treatment
0,742	0,004	UGT2B substrate
0,734	0,005	MMP9 expression inhibitor
0,729	0,002	Astringent
0,735	0,014	CYP2C substrate
0,730	0,010	27-Hydroxycholesterol 7alpha-monooxygenase inhibitor
0,751	0,034	Mucomembranous protector
0,721	0,004	Malate dehydrogenase inhibitor
0,719	0,003	Interleukin 4 antagonist
0,720	0,004	Histamine release inhibitor
0,717	0,006	Chemopreventive
0,708	0,004	UGT2B12 substrate
0,706	0,006	CYP2A4 substrate
0,706	0,007	Hepatoprotectant
0,704	0,015	Antiinflammatory
0,714	0,049	Gluconate 2-dehydrogenase (acceptor) inhibitor
0,714	0,050	Testosterone 17beta-dehydrogenase (NADP+) inhibitor
0,721	0,049	CDP-glycerol glycerophosphotransferase inhibitor
